# TRAP1 ablation improves mitochondrial cristae and oxidative phosphorylation in pancreatic cancer stem cells

**DOI:** 10.20517/cdr.2025.229

**Published:** 2026-05-15

**Authors:** Giulia Ambrosini, Elisa Dalla Pozza, Ilaria Cristanini, Sara Vinco, Enrica Cappellozza, Barbara Cisterna, Claudio Laquatra, Andrea Rasola, Emanuela Bottani, Ilaria Dando

**Affiliations:** ^1^Department of Neurosciences, Biomedicine and Movement Sciences, University of Verona, Verona 37134, Italy.; ^2^Department of Diagnostics and Public Health, University of Verona, Verona 37134, Italy.; ^3^Department of Biomedical Sciences, University of Padova, Padova 35131, Italy.

**Keywords:** Cancer stem cells, pancreatic ductal adenocarcinoma, TRAP1, CDH1, mitochondria, oxidative phosphorylation

## Abstract

**Aim:** Cancer stem cells (CSCs) in pancreatic ductal adenocarcinoma (PDAC) display high metabolic plasticity, supporting tumor aggressiveness and therapeutic resistance. Here, we investigated the role of the mitochondrial chaperone TRAP1 in regulating mitochondrial architecture, metabolism, and adhesion in CSCs.

**Methods:** We studied an *in vitro* model of CSCs using Panc1 cells and the corresponding stable TRAP1-knockout cells (TRAP1-KO). Molecular techniques used were quantitative polymerase chain reaction (qPCR), Western blot, transmission electron microscopy, and Seahorse technology.

**Results:** CSCs showed increased TRAP1 expression after 2 weeks of culture, reflecting a preferential metabolic shift toward glycolysis. TRAP1 deletion impaired the ability of CSCs to form compact spheroids without altering canonical CSC traits, such as reduced proliferation, increased stem marker expression, and enhanced chemoresistance. We demonstrate that TRAP1 deletion increases *CDH1*, an effect that was reversed by succinate supplementation, indicating that the TRAP1–succinate-CDH1 axis controls adhesion-related properties. Ultrastructural analyses revealed profound mitochondrial remodeling in the absence of TRAP1: parental cells displayed enlarged, elongated mitochondria with wider cristae, whereas CSCs developed fragmented mitochondria with thinner cristae and tighter crista junctions. These alterations were closely associated with the differential regulation of mitochondrial fission factor (MFF). Functionally, loss of TRAP1 enhanced oxidative phosphorylation, leading to increased mitochondrial adenosine triphosphate (ATP) production, elevated maximal respiration, and reduced proton leak.

**Conclusion:** Collectively, these findings identify TRAP1 as a critical regulator of mitochondrial organization, respiratory efficiency, and *CDH1*-mediated adhesion in PDAC CSCs, highlighting metabolic and structural vulnerabilities that may be exploited therapeutically to destabilize CSC homeostasis and enhance treatment response.

## INTRODUCTION

Pancreatic ductal adenocarcinoma (PDAC) remains one of the most aggressive malignancies with minimal treatment options. With a 5-year survival rate of 12%, it is currently one of the leading causes of cancer death worldwide^[1]^. Due to the lack of adequate screening methods, late symptom onset, and high resistance to standard therapies, the prognosis for this malignancy remains poor. Indeed, fewer than 20% of patients present with a resectable tumor mass at the time of initial diagnosis^[2]^.

From a biochemical perspective, PDAC cells exhibit extensive metabolic reprogramming to meet the energetic demands generated by the extremely harsh conditions of their microenvironment. Malignant progression of PDAC, from precancerous lesions to advanced invasive and metastatic stages is driven by the activation of oncogenes such as *KRAS* and the inactivation of several tumor suppressor genes, including *p16/CDKN2A*, *TP53*, and *DPC4/SMAD4*^[3]^. The highly chemoresistant and metastatic nature of this tumor is linked to a subpopulation of highly plastic stem-like cells within the tumor, known as cancer stem cells (CSCs)^[4]^. CSCs represent the principal source of tumor initiation, metastasis, and cellular heterogeneity, as they adapt to diverse stimuli and microenvironmental cues^[5,6]^.

Over the past few decades, cancer treatment strategies have evolved from conventional radiation and chemotherapy to recent targeted therapies and immunotherapy. The discovery of CSCs provided one of the earliest indications of tumor heterogeneity and CSC eradication has emerged as a critical goal for preventing tumor relapse and therapy resistance. Since one of the defining features of the CSC phenotype is its plastic ability to rewire metabolism^[4]^, targeting metabolic pathways selectively deregulated in CSCs is a promising therapeutic strategy, and several clinical trials are ongoing. However, the vulnerabilities of different tumor types to specific inhibitors, either as single agents or in combination with standard treatments, remain to be fully elucidated^[7]^.

TRAP1, also dubbed heat shock protein 75 (HSP75)^[8]^, is the mitochondrial paralog of heat shock protein 90 (HSP90). Recent reports demonstrate that TRAP1 is involved in the metabolic rewiring of tumor cells^[9]^ by tuning key pathways, including oxidative phosphorylation (OXPHOS) through the inhibition of electron transport chain complexes^[10-12]^. TRAP1 expression is higher in many tumors compared to surrounding non-malignant tissues^[13]^. Furthermore, its protein levels correlate with malignant progression and metastasis in several neoplastic models^[14]^, including pancreatic cancer^[15]^. This makes TRAP1 a promising target for anticancer drugs development^[14]^. We previously demonstrated that CSCs possess the ability to dynamically modify their metabolic features during progressive dedifferentiation. They switch from a glycolytic metabolism to one mainly relying on OXPHOS, reaching a dormant metabolic state at the apex of stemness^[4,16]^. Here, we investigated for the first time the expression levels of TRAP1 in an *in vitro* model of PDAC CSCs and characterizes its role in the metabolic organization of these cells.

## METHODS

### Drugs and chemicals

Gemcitabine (Sandoz S.p.a), Irinotecan (Fresenius Kabi Italia S.r.l.), and Oxaliplatin (Accord Healthcare S.L.U.) were stored at 4 °C, and 5-fluorouracil (Teva Italia S.r.l.) was at room temperature (RT). Sodium succinate hexahydrate was was dissolved in phosphate-buffered saline (PBS; Thermo Scientific).

### Cell lines

The pancreatic adenocarcinoma cell lines Panc1, MiaPaCa2, HS776T, Suit2, and PaCa3 were grown in RPMI-1640 supplemented with 10% fetal bovine serum (FBS) and 50 µg/mL gentamicin sulfate (all from Gibco/Life Technologies, USA) and were maintained at 37 °C with 5% CO_2_. CSCs were obtained as previously described^[4]^. Briefly, adherent cells were washed twice in 1× PBS (Gibco/Life Technologies, USA) and then cultured in stem-specific medium, i.e., DMEM/F-12 without glucose (US Biological Life Sciences, USA) supplemented with 1 g/L glucose, B27, 1 µg/mL fungizone, 1% penicillin/streptomycin (all from Gibco/Life Technologies, USA), 5 µg/mL heparin (Sigma/Merck), 20 ng/mL epidermal growth factor (EGF), and 20 ng/mL fibroblast growth factor (FGF) (both from PeproTech, United Kingdom). Panc1 cells were cultured in flasks with a hydrophobic surface specifically designated for the growth of suspension cells and were maintained at 37 °C with 5% CO_2_ in the stem-specific medium 8 weeks (8 w), refreshed twice a week with new medium. Before each experiment, cells were passed through a cell strainer (40 µm) to separate and maintain only the cell aggregates/spheres, which were trypsinized to obtain single-cell suspension. Bright-field images were acquired with an inverted microscope (Axio Vert. A1, Zeiss, Germany). In all the assays, both parental cells and CSCs were cultured in proliferating conditions and did not undergo contact inhibition–induced growth arrest.

The human pancreatic cancer cell line Panc1, along with the other cell lines used in this study (MiaPaCa2, HS776T, Suit2, and PaCa3) were previously purchased from American Type Culture Collection (ATCC) or kindly provideded by colleagues at University of Verona, Italy. Ethical approval was not required for this study as all cell lines were commercially obtained or provided by collaborators.

### CRISPR-Cas9 technology

TRAP1-knockout Panc1 cell line was generated as previously reported by Cannino *et al.*^[12]^. In brief, guide RNAs specific for human TRAP1 were annealed and cloned into the lentiCRISPRv2 vector (Addgene plasmid #52961). The construct was co-transfected into HEK293T cells together with the packaging plasmids pMDLg/pRRE, pRSV-Rev, and pMD2.G to produce lentiviral particles. These recombinant lentiviruses were subsequently used to transduce Panc1 cells, and stable knockout cells were selected using 1 µg/mL puromycin.

### RNA extraction and quantitative polymerase chain reaction

Total RNA was extracted from about 1 × 10^6^ cells using TRIzol Reagent (Life Technologies, USA) and 1 µg of RNA was using a first-strand cDNA synthesis kit. Real-time quantification was performed in duplicate samples by SYBR-Green detection chemistry with GoTaq quantitative polymerase chain reaction (qPCR) Master Mix (Promega, USA) on a QuantStudio 3 Real-Time PCR System (Thermo Fisher Scientific, USA). The primers used were listed in Table 1.

**Table 1:** qPCR primers’ list

**Target gene**	**Primer sequences (5’-3’)**
*CD29* forward	GACGCCGCGCGGAAAA
*CD29* reverse	ACATCGTGCAGAAGTAGGCA
*CD44* forward	AGAAGGTGTGGGCAGAAGAA
*CD44* reverse	AAATGCACCATTTCCTGAGA
*CDH1* forward	GACACCAACGATAATCCTCCGA
*CDH1* reverse	GGCACCTGACCCTTGTACGT
*CDH3* forward	TCCTTCTCCAGGTTTGCTGG
*CDH3* reverse	GAATACTTTCCCCAGCGCCT
*E-selectin* forward	AGTCCTCTTGTGCCTTCAGC
*E-selectin* reverse	GATCTTTCCCGGAACTGCCA
*NANOG* forward	AGTCCCAAAGGCAAACAACCCACTTC
*NANOG* reverse	TGCTGGAGGCTGAGGTATTTCTGTCTC
*MUC-4* forward	TCAATGGTGGTCGTGTGATT
*MUC-4* reverse	AAGTCGGTGCAGCTGTCTCT
*MUC-16* forward	GATGTCAAGCCAGGCAGCACAA
*MUC-16* reverse	GAGAGTGGTAGACATTTCTGGGC
*OCT3/4* forward	GACAGGGGGAGGGGAGGAGCTAGG
*OCT3/4* reverse	CTTCCCTCCAACCAGTTGCCCCAAAC
*SOX2* forward	GGGAAATGGGAGGGGTGCAAAAGAGG
*SOX2* reverse	TTGCGTGAGTGTGGATGGGATTGGTG
*SDHA* forward	GGACCTGGTTGTCTTTGGTC
*SDHA* reverse	CCAGCGTTTGGTTTAATTGG
*ZEB1* forward	GTTACCAGGGAGGAGCAGTGAAA
*ZEB1* reverse	GACAGCAGTGTCTTGTTGTTGTAGAAA

qPCR: Quantitative polymerase chain reaction.

The cycling conditions used were: 95 °C for 2 min, 40 cycles at 95 °C for 3 s, 60 °C for 30 s, 95 °C for 15 s, and 60 °C for 1 min. The average of cycle threshold of each triplicate was analyzed according to the 2^-∆∆Ct^ method using *SDHA* as an endogenous control.

CDH1 levels were also analyzed following treatment with succinate. Cells were seeded in 60-mm Ø plates (3.5 × 10^5^ cells/plate) and, the day after, were treated for 48 h with succinate at different concentrations: 0.5, 2, and 20 mM.

### Cell proliferation assay

Panc1 parental cells (P), TRAP1-KO cells, and CSCs at 2, 4, and 8 weeks of culture were plated in 24-well cell culture plates (3 × 10^4^ cells/well) in their respective medium and were incubated at 37 °C with 5% CO_2_. Viable cells were counted by Trypan Blue dye exclusion after 2, 4, 7, and 10 days of culture. The doubling time was calculated using the formula: T = (T_2_ - T_1_) × log2/log(Q_2_/Q_1_), where: T_1_, day 0; T_2_, day 10; Q_1_, cell number at day 0; and Q_2_, cell number at day 10.

To test the cell viability after treatment, cells were seeded in 96-well plates (5 × 10^3^ cells/well) and, on the following day, were incubated with compounds at the following concentrations: 50 µM Gemcitabine, 100 µM Oxaliplatin, 200 µM Irinotecan, and 1 mM 5-fluorouracil. At the end of the treatment (48 h for all compounds), cell growth was measured by Resazurin assay (Immunological Sciences, Italy) according to the manufacturer’s protocol and fluorescence was measured by Tecan Infinite 200 PRO microplate reader (Ex: 535 nm, Em: 590 nm).

### Protein extraction and immunoblotting

To prepare the samples, frozen cell pellets were resuspended in lysis buffer {i.e., 1 mM Na_3_VO_4_, 1 mM NaF, 2 mM ethylenediaminetetraacetic acid (EDTA), 0.2 mM phenylmethylsulfonyl fluoride (PMSF), 150 mM NaCl, 1× complete protease inhibitor cocktail, and radioimmunoprecipitation assay (RIPA) buffer pH 8.0 [150 mM NaCl, 50 mM Tris-HCl, 1% Igepal, 0.5% sodium deoxycholate, and 0.1% sodium dodecyl sulfate (SDS)]}. The lysate was centrifuged at 12,000 × *g* for 10 min at 4 °C and the supernatant was used for immunoblotting. Protein concentration was measured with the Bradford Reagent (SERVA electrophoresis, Heidelberg, Germany) using bovine serum albumin (BSA) as a standard. Thirty µg of protein suspended in SDS loading buffer were run on 12% SDS polyacrylamide gels and electrotransferred to polyvinylidene difluoride (PVDF) membranes (Merck Millipore, Burlington, MA, USA). Membranes were then incubated for 1 h at RT with blocking solution, i.e., 5% nonfatdry milk in tris-buffered saline with Tween 20 (TBST) pH 7.5 (100 mM Tris-HCl, 0.1% Tween-20, and 0.9% NaCl). Then, the membranes were incubated with primary antibodies at appropriate dilutions in blocking solution overnight at 4 °C. Primary antibodies included the following: TRAP1 (1:1,000, sc-73604 Santa Cruz Biotechnology), E-cadherin (1:1,000, #A20798 ABclonal), phospho-DRP1 (p-DRP1; 1:1,000, #4494), dynamin-related protein 1 (DRP1; 1:1,000, #8570), mitochondrial fission factor (MFF; 1:1,000, #E5W4M), optic atrophy 1 (OPA-1; 1:1,000, #80471), mitofusin 1 (MFN1; 1:1,000, #14739), and mitofusin 2 (MFN2; 1:1,000, #11925). All antibodies were obtained from Cell Signaling Technology unless otherwise specified. Horseradish-peroxidase-conjugated anti-mouse (1:8,000; KPL #074-1806) or anti-rabbit (1:10,000; KPL #074-1516) were used as secondary antibodies. The immunocomplexes were visualized by chemiluminescence using the ChemiDoc MP Imaging System (Bio-Rad Laboratories, Hercules, CA, USA), and the intensity of the chemiluminescence response was measured by processing the image using NIH ImageJ software (http://rsb.info.nih.gov/nih-image/). Amido black staining was used to confirm loading in different lanes.

### Transmission electron microscopy

Panc1 P, TRAP1-KO cells, CSCs 2w, and CSCs 2w TRAP1-KO were processed for the ultrastructural morphological and morphometric evaluation of mitochondria by transmission electron microscopy (TEM). Cell samples were fixed with 2.5% glutaraldehyde and 2% paraformaldehyde in 0.1 M phosphate buffer, pH 7.4, at 4 °C for 2 h. Cell samples were then rinsed in PBS, postfixed with 1% OsO_4_ and 1.5% potassium ferrocyanide for 1 h at 4 °C, dehydrated with acetone, and embedded in Epon 812 resin. Ultrathin sections (80-nm thick) were stained with lead citrate for 1 min and observed with a Philips Morgagni transmission electron microscope operating at 80 kV and equipped with a Megaview III camera for digital image acquisition. Morphometric evaluation of the mitochondrial area, aspect ratio, cristae extension, cristae width, and cristae junction diameter were performed. Micrographs (×18,000) of 50 randomly selected mitochondria from 20 different cells were taken and the area and the lengths of the major and the minor mitochondrial axes were measured. The aspect ratio parameter (index of mitochondrial elongation) was calculated as the ratio of the two lengths. The length of the outer and the inner mitochondrial membrane was measured (micrographs at ×36,000) in 30 mitochondria per sample, and the inner/outer membrane ratio was calculated as an assessment of the cristae extension independent of mitochondrial size. Cristae width and junction diameter were measured in 15 mitochondria for each sample (micrographs at ×44,000).

The measurements were performed using ImageJ software (NIH Image, Bethesda, MD, USA). The means ± standard error (SE) were calculated for all of the mitochondrial parameters and the statistical comparison was performed with the Kruskal–Wallis non-parametric test followed by the Mann–Whitney test for pairwise comparison. Statistical significance was set at *P* ≤ 0.05.

### Seahorse extracellular flux analysis

Extracellular oxygen consumption rate (OCR) and extracellular acidification rate (ECAR) were measured with the Seahorse XF24 Extracellular Flux Analyzer (Seahorse Bioscience, USA).

Panc1 P cells, Panc1 TRAP1-KO P cells (3 × 10^4^ cells/well), and CSCs 2w TRAP1-KO (6 × 10^4^ cells/well), were plated in the V7 XFe-24-well cell culture microplate precoated with 3 mg/mL collagen I (Gibco/Life Technologies, USA) and cultured at 37 °C with 5% CO_2_ overnight to reach the appropriate confluence. On the day of the assay, cells were incubated in Seahorse XF DMEM Medium (Seahorse Bioscience, cat. No. 103575-100) supplemented with 10 mM glucose, 1 mM pyruvate, 2 mM glutamine, pH 7.4, for 1 h in a non-CO_2_ incubator.

For real-time adenosine triphosphate (ATP) rate assay, OCR, and ECAR were recorded at the baseline and after sequentially adding oligomycin (ATP synthase inhibitor; 1 µM) through port A, and a combination of rotenone (complex I inhibitor; 1 µM), and antimycin A (complex III inhibitor; 1 µM) through port B.

For Mito stress test OCR and ECAR were recorded at the baseline and after sequentially adding oligomycin A (1 µM; port A), Carbonyl cyanide 4-(trifluoromethoxy) phenylhydrazone (FCCP, 1.5 μM; port B), and rotenone/antimycin A (1 µM / 1 µM; port C).

Raw OCR and ECAR were normalized to the DNA content per well which was quantified with the CyQUANT Cell proliferation assay kit (Thermo Fisher Scientific, Cat. No. C35007), according to the manufacturer’s instructions, on a Victor X4 multilabel microplate reader (Perkin Elmer). Real-time ATP rate assay results were expressed as the calculated percentage contribution of glycolysis and OXPHOS to total ATP production, as well as the ratio between mitochondrial and glycolytic ATP production rates. Mito Stress Test results were expressed as fold change relative to the parental (P) cell line. Data from at least three independent biological experiments were reported and statistically analyzed.

### Statistical analysis

Results are presented as mean ± SE of at least three biological replicates. Statistical significance was determined using two-tailed Student’s *t*-test or one-way analysis of variance (ANOVA) for multiple comparisons, as appropriate. When ANOVA was performed, Tukey’s post hoc test was used for pairwise comparisons. Data analyses were carried out using GraphPad Prism software (version 7.0), and statistical significance was defined as *P* < 0.05.

## RESULTS

### CSCs express higher levels of TRAP1 than parental cells

**Figure 1:**
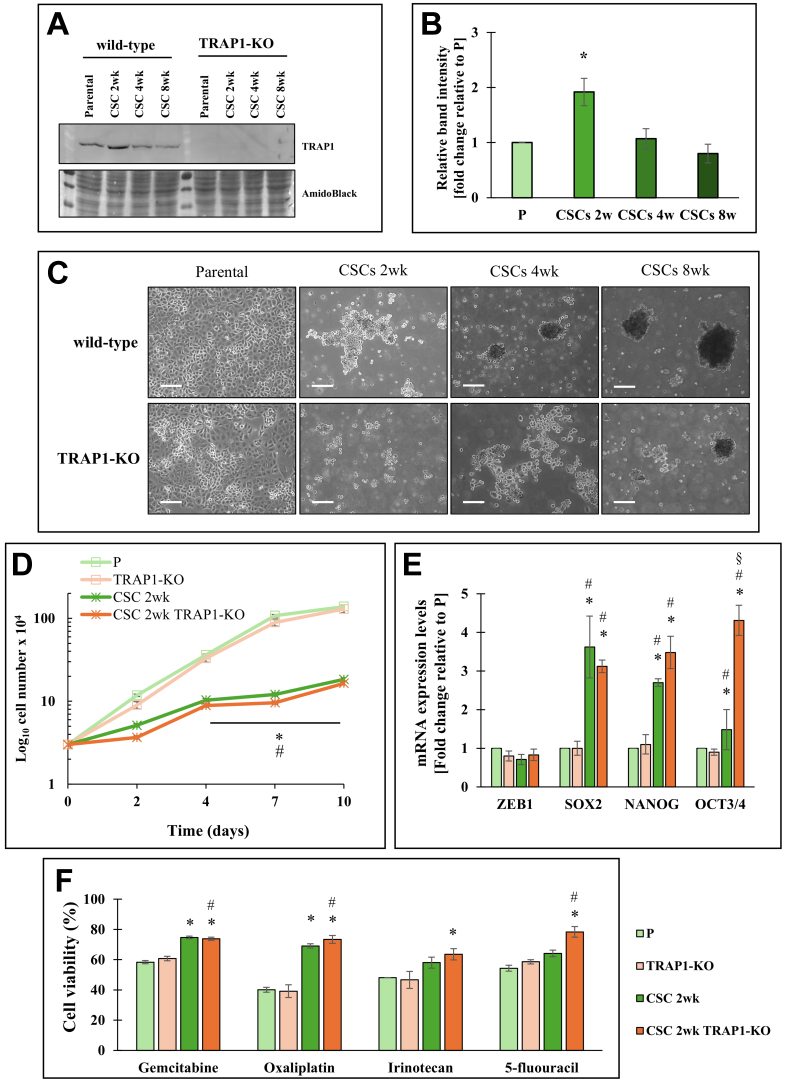
Effects of TRAP1 in Panc1 cells and derived CSCs. (A) Representative Western blot of TRAP1 expression in Panc1 parental cells (P), CSCs at different cell culture times (2, 4, 8 weeks) and TRAP1-Knock out (TRAP1-KO) cells and the derived CSCs. Amido black is shown as the loading control; (B) The histogram represents the average of band intensity quantification of three independent biological replicates and is reported as fold change relative to P cells; (C) Bright field representative images of Panc1 parental cells, CSCs at different cell culture times (2, 4, 8 weeks) and TRAP1-KO cells and the derived CSCs. All the pictures were taken with 5× magnification. Scale bar: 50 μm; (D) Proliferation rate of P cells, CSCs 2w, TRAP1-KO cells, and CSCs 2w TRAP1-KO, in which 3 × 10^4^ cells/well were plated for each condition at time 0 and counted after the indicated days; (E) qPCR analysis of stemness genes (*ZEB1*, *SOX2*, *NANOG*, *OCT3/4*) in P cells, CSCs 2w, TRAP1-KO cells, and CSCs 2w TRAP1-KO; (F) Cell viability analysis of P cells, CSCs 2w, TRAP1-KO cells, and CSCs 2w TRAP1-KO treated with 50 μM gemcitabine or 100 μM Oxaliplatin or 200 μM Irinotecan or 1 mM 5-fluorouracil for 48 h. The values are reported as fold change relative to P cells. All values are the means (± SE) of at least three independent biological replicates. Statistical legend: ^*^P < 0.05 indicated condition *vs.* P cells, ^#^condition *vs.* TRAP1-KO, ^§^condition *vs.* CSCs 2w. Statistical methods: two-tailed Student’s *t* test. CSCs: Cancer stem cells; qPCR: quantitative polymerase chain reaction; SE: standard error.

TRAP1 is emerging as a crucial target for cancer therapy, due to its expression in diverse neoplastic tissues, together with its expression in different cancer cell lines, including PDAC cell models [Supplementary Figure 1A]. We evaluated TRAP1 protein expression by Western blotting in five different PDAC cell lines (MiaPaca2, Panc1, HS776T, Suit2, and PaCa3; Supplementary Figure 1B). While all cell lines displayed TRAP1 expression, Suit2 cells exhibited the highest level. However, as Suit2 cells failed to generate well-dedifferentiated CSCs in our models, we focused our analysis on Panc1 cells, the most thoroughly characterized CSC model in our laboratory. To investigate the role of TRAP1 in Panc1-derived CSCs, we analyzed its protein expression during progressive de-differentiation at three time points: 2 weeks (2 w), 4 weeks (4 w), and 8 weeks (8 w) of culture in the specific stem culture medium. We have previously shown that these time points display a progressive stem phenotype and different metabolic properties^[4,16]^. After 2 weeks in culture, CSCs exhibit increased glycolytic activity^[4]^ and increased TRAP1 expression levels compared to both parental (P) cells [Figure 1A and B]. Based on these findings, we stably knocked out TRAP1 in Panc1 P cells and subsequently derived the relative CSCs [Figure 1A]. Both P cell types (with or without TRAP1) exhibited a similar morphology. However, TRAP1-KO CSCs generated smaller and fewer spheroids, characterized by less defined borders than their wild-type counterparts [Figure 1C]. These differences were observed up to 8 weeks in culture, indicating a long-lasting effect of TRAP1 on the CSC phenotype. Given that TRAP1 expression is maximally induced after 2 weeks in culture, we focused on this time point its role in modulating stemness.

Analysis of cell proliferation [Figure 1D and Supplementary Table 1], stem marker expression (ZEB1, SOX2, NANOG, and OCT3/4; Figure 1E), and cell viability after exposure to four different chemotherapy agents [Figure 1F] showed no differences between P cells and CSCs, regardless of TRAP1 expression. Nonetheless, TRAP1-KO CSCs had a slower proliferative capacity, an increase in stem marker expression, and increased chemotherapy resistance compared to parental cells.

### TRAP1 regulates CDH1 expression through succinate levels

**Figure 2:**
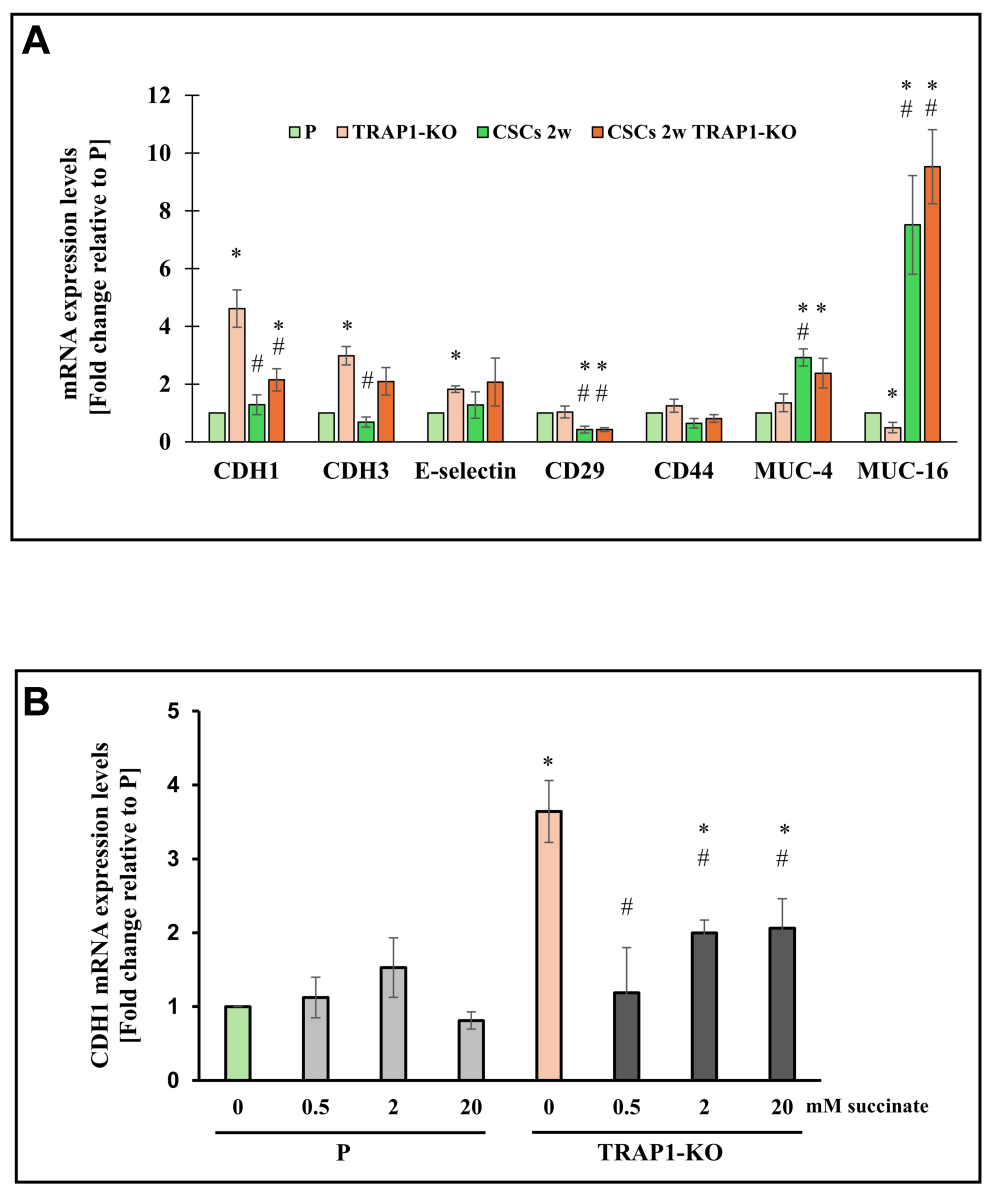
TRAP1 affects adhesion molecule expression in Panc1 cells and derived CSCs. (A) qPCR analysis of adhesion molecules (*CDH1*, *CDH3*, *E-selectin*, *CD29*, *CD44*, *MUC-4* and *MUC-16*) in P cells, CSCs 2w, TRAP1-KO cells, and CSCs 2w TRAP1-KO; (B) qPCR analysis of *CDH1* expression in P or TRAP1-KO cells treated with different doses of succinate for 48 h. All values are reported as fold change relative to P cells and are the means (± SE) of at least three independent biological replicates. Statistical legend: ^*^P < 0.05 indicated condition *vs.* P cells, ^#^condition *vs.* TRAP1-KO. Statistical methods: two-tailed Student’s *t*-test. CSCs: Cancer stem cells; qPCR: quantitative polymerase chain reaction; SE: standard error; mRNA: messenger RNA.

As TRAP1-KO CSCs were unable to form spheroids, we analyzed the messenger RNA (mRNA) levels of various genes involved in cell adhesion, such as *CD44*, *CD29*, *MUC-4*, *MUC-16*, *CDH1*, *CDH3*, and *E-selectin*. Among these, *CD44* expression remained unchanged. In contrast, *CD29*, *MUC-4*, and *MUC-16* were regulated similarly in both wild-type and TRAP1-KO CSCs compared to P cells; specifically, *CD29* expression decreased while *MUC-4* and *MUC-16* increased. This suggests that their modulation depends primarily on the acquisition of stem properties [Figure 2A]. Notably, TRAP1 ablation increased the expression of *CDH1*, *CDH3*, and *E-selectin* in P cells [Figure 2A].

The *CDH1* gene, which encodes E-cadherin, deserves further consideration. Cells lacking TRAP1 expression upregulated both *CDH1* mRNA [Figure 2A] and E-cadherin protein levels [Supplementary Figure 2A]. In addition, CDH1 mRNA expression was low in Suit2 cells [Supplementary Figure 2B], supporting an inverse correlation between TRAP1 levels and *CDH1* expression.

Previous studies have reported that TRAP1 downregulates the enzymatic activity of succinate dehydrogenase, leading to the accumulation of the oncometabolite succinate^[17]^. Therefore, we analyzed whether *CDH1* expression is regulated in a succinate-dependent manner. We treated both P and TRAP1-KO cells with varying concentrations of succinate and analyzed *CDH1* expression levels. As shown in Figure 2B, the expression levels of *CDH1* decreased significantly in Panc1 TRAP1-KO cells treated with succinate, while no changes in *CDH1* expression were observed in TRAP1-expressing P cells. These data indicate that TRAP1 decreases *CDH1* expression via a succinate-dependent mechanism.

### TRAP1 regulates mitochondria shape and their internal organization

**Figure 3:**
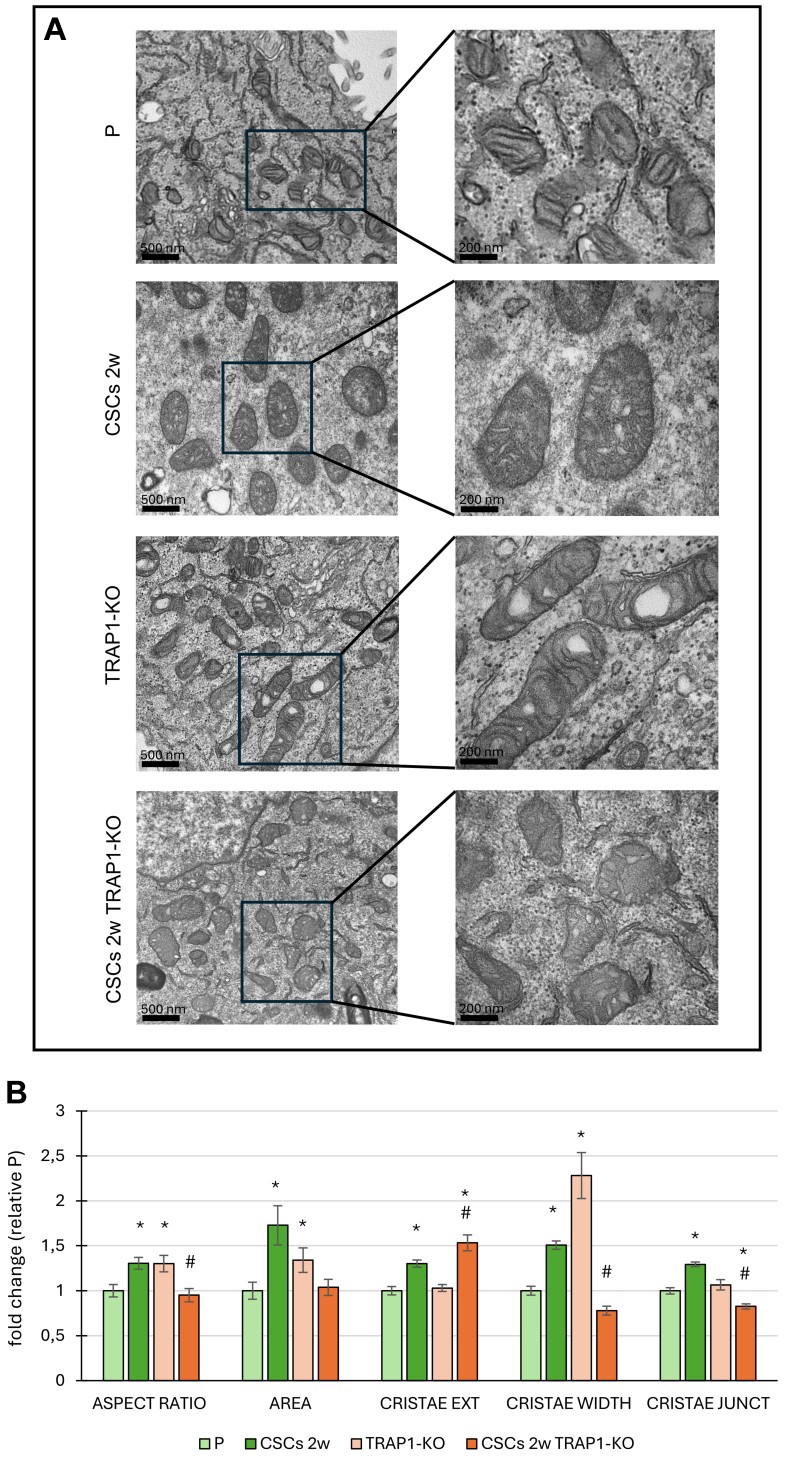
TRAP1 affect mitochondrial morphology in Panc1 cells and derived CSCs. (A) Representative images of mitochondria of P cells, CSCs 2w, TRAP1-KO cells, and CSCs 2w TRAP1-KO analyzed by TEM. Scale bars are reported in the figure and correspond to 500 nm on the left panels and 200 nm on the right panels; (B) Histograms represent parameter analysis derived from TEM images of aspect ratio and area of mitochondria, and extension, width, junction dimension of mitochondrial cristae. Values are reported as fold change relative to P cells (± SE). Statistical legend: ^*^P < 0.05 indicated condition *vs.* P cells, ^#^condition *vs.* TRAP1-KO. Statistical methods: two-tailed Student’s *t*-test. CSCs: Cancer stem cells; TEM: transmission electron microscopy; SE: standard error.

Since TRAP1 is a molecular chaperone involved in maintaining mitochondrial homeostasis^[9]^, we investigated whether mitochondrial morphology, which is closely linked to functional activity^[18]^, undergoes TRAP1-dependent changes. Transmission electron microscope (TEM) analysis revealed that mitochondria undergo extensive structure remodeling in P cells in the absence of TRAP1 [Figure 3A]. Specifically, TRAP1-KO mitochondria exhibit a significant increase in area, aspect ratio (major-to-minor axis length ratio), and cristae width [Figure 3B].

In dedifferentiated cells, the mitochondria of TRAP1-KO CSCs showed greater cristae extension and reduced crista junction diameter compared with those of P cells. Notably, a comparison between P cells and CSCs, both lacking TRAP1, further confirms these differences, alongside a significantly lower aspect ratio and cristae width [Figure 3B].

**Figure 4:**
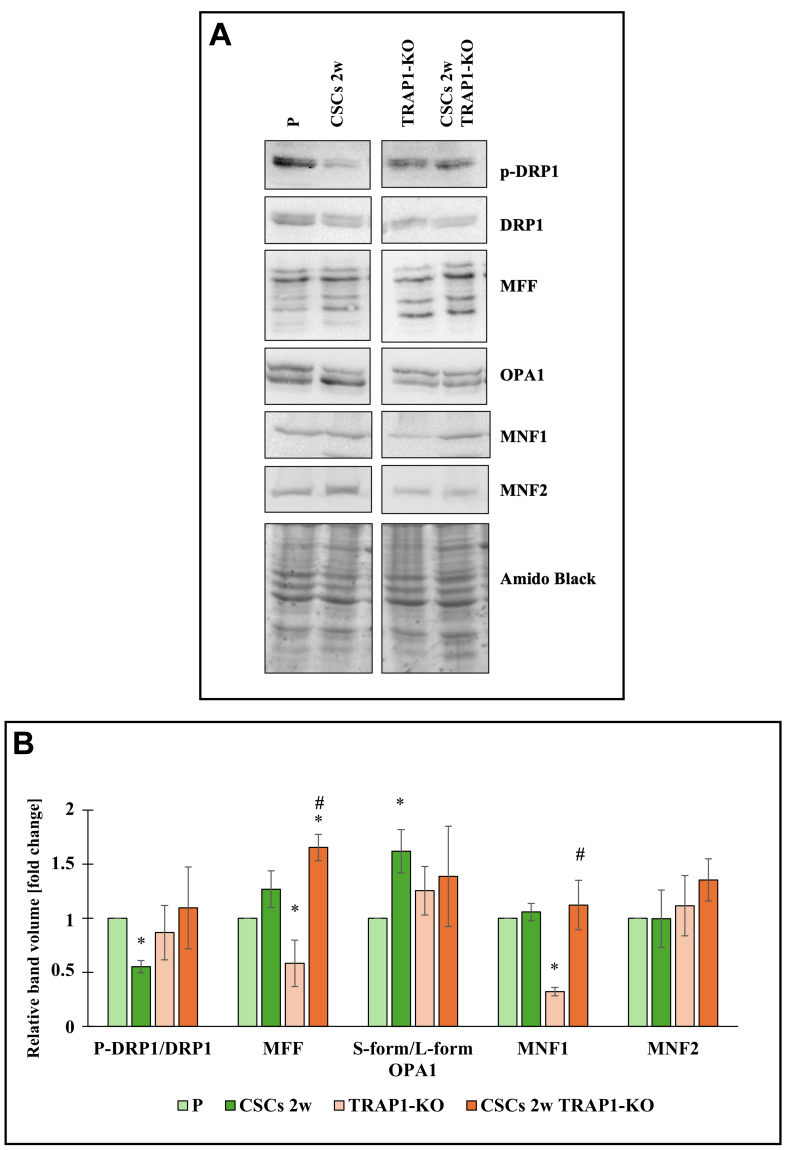
TRAP1 regulates mitochondrial dynamics proteins in Panc1 cells and derived CSCs. (A) Representative Western blot of expression of mitochondrial dynamics proteins (pDRP1, DRP1, MFF, OPA-1, MFN1, and MFN2) in P cells, CSCs 2w, TRAP1-KO cells, and CSCs 2w TRAP1-KO. Amido black is shown as the loading control; (B) The histogram represents the quantification of band volume reported as fold change relative to P cells. All values are reported as fold change relative to P cells and are the means (± SE) of at least three independent biological replicates. Statistical legend: ^*^P < 0.05 indicated condition *vs.* P cells, ^#^condition *vs.* TRAP1-KO. Statistical methods: two-tailed Student’s *t*-test. CSCs: Cancer stem cells; p-DRP1: phospho-dynamin-related protein 1; MFF: mitochondrial fission factor; OPA-1: optic atrophy 1; MFN1: mitofusin 1; MFN2: mitofusin 2; SE: standard error.

From a molecular perspective, mitochondrial dynamics is tightly controlled by specific proteins, such as DRP1 and MFF that regulate mitochondrial fission, and OPA1, MFN1, and MFN2 that elicit mitochondrial fusion^[19]^. As reported in Figure 4 and consistent with our previous work^[16]^, the expression of the active form of DRP1 (p-DRP1) is decreased in TRAP1-expressing CSCs compared to their parental counterpart. This is in line with the increased levels of OPA1 observed in these cells (Figure 4B, green histograms). In contrast, when TRAP1 is deleted, the expression of these proteins remains unchanged, whereas MFF is strongly down-regulated in TRAP1-KO parental cells. Conversely, MFF expression is strongly increased in TRAP1-KO CSCs after 2 weeks in culture (Figure 4B, orange histograms), corroborating the TEM observations. Regarding the other proteins involved in mitochondrial fusion, Figure 4 shows that MFN1 is regulated only in TRAP1-KO cells, whereas MFN2 levels remain consistent among all cell types. Altogether, these data show that ablation of TRAP1 in parental cells is associated with more elongated mitochondria that present larger cristae, whereas the absence of TRAP1 in CSCs prompts mitochondria fragmentation and extension of tightly connected cristae, consistent with MFF upregulation.

### TRAP1 ablation increases OXPHOS in P cells and CSCs

**Figure 5:**
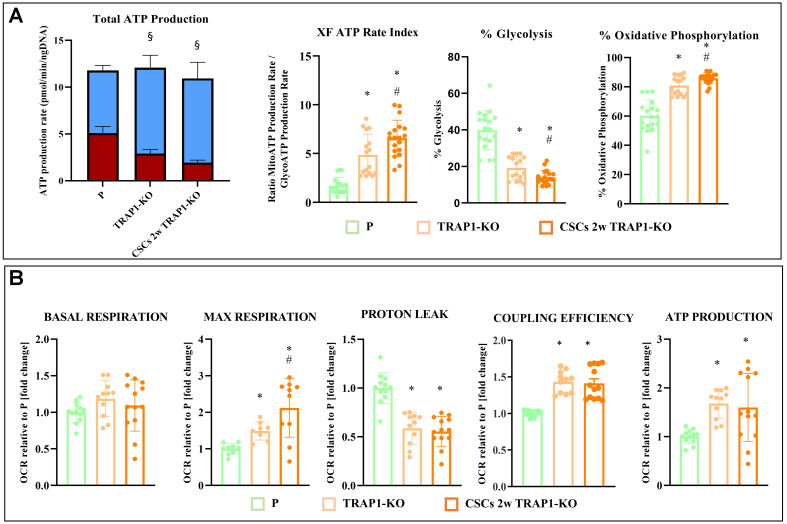
TRAP1 regulates metabolic pathway in Panc1 cells and derived CSCs. (A) Real-time ATP rate assay with Seahorse technology in P cells, CSCs 2w, TRAP1-KO cells, and CSCs 2w TRAP1-KO. ATP produced by glycolysis (GlycoATP) is shown in red, whereas ATP produced by OXPHOS (MitoATP) is in blue; (B) OCR with Seahorse technology in P cells, CSCs 2w, TRAP1-KO cells, and CSCs 2w TRAP1-KO. All values are reported as fold change relative to P cells and are the means (± SE) of at least three independent biological replicates. Statistical legend: ^*^P < 0.05 indicated condition *vs.* P cells, ^#^condition *vs.* TRAP1-KO, ^§^GlycoATP *vs.* MitoATP in the same cellular condition. Statistical methods: one-way ANOVA. CSCs: Cancer stem cells; ATP: adenosine triphosphate; OXPHOS: oxidative phosphorylation; OCR: oxygen consumption rate; SE: standard error.

To evaluate the effect of TRAP1 on cell bioenergetics, we analyzed the ATP production rates from distinct metabolic pathways. While total ATP production rate remained unchanged across P cells, TRAP1-KO cells, and CSCs 2w TRAP1-KO, the energetic source shifted significantly towards OXPHOS utilization when TRAP1 was knocked out [Figure 5A, Supplementary Figure 3A and B]. Indeed, TRAP1 ablation in P cells significantly decreased the glycolytic ATP production rate while enhancing OXPHOS-derived ATP; this effect was further enhanced in de-differentiated TRAP1-KO CSCs [Figure 5A]. These data suggest that TRAP1 loss increases mitochondrial respiration, while limiting the glycolytic flux. To validate this metabolic rewiring, we assessed mitochondrial OXPHOS using a standard Mitostress Test [Figure 5B and Supplementary Figure 3C]. Despite comparable basal respiration, the mitochondrial ATP-linked respiration was significantly higher in both P and CSC TRAP1-KO cells. Moreover, both TRAP1-KO cell lines exhibited a stronger FCCP-stimulated increase in maximal respiration, indicating an enhanced ability to utilize mitochondrial oxidative capacity under high energetic demand. Notably, this effect was significantly more pronounced in TRAP1-KO CSCs compared to TRAP1-KO P cells, correlating with the thicker cristae observed by TEM in the former cells [Figure 3B]. Finally, the reduced proton leak and higher coupling efficiency in TRAP1-deficient cells [Figure 5B] further support a more tightly coupled mitochondrial state and a lower propensity for mitochondrial membrane potential dissipation.

## DISCUSSION

The presence of CSCs in the tumor mass denotes aggressiveness and metastatic potential^[6]^, largely due to their ability to modify and flexibly adapt their metabolic requirements to the fluctuating environmental conditions^[4]^. Understanding the unique metabolic features of CSCs is crucial for defining new potential therapeutic strategies against them. In this study, we investigated for the first time the role of the molecular chaperone TRAP1 in an *in vitro* model of pancreatic CSCs. TRAP1 decreases the activity of electron transport chain complexes^[10,11]^, thereby altering the mitochondrial metabolic flux. Our findings reveal that TRAP1 is more than a protein whose expression is tuned during dedifferentiation. Indeed, it represents a key regulator of CSC phenotype, influencing both mitochondrial cristae morphology and metabolic activity, as well as the CSC adhesion properties.

Using a progressive de-differentiation model of CSCs derived from the Panc1 cell line, we found that TRAP1 expression was twofold higher in short-term cultured CSCs (CSCs 2w) than in parental cells. This finding is consistent with our previous reports showing that CSCs at this stage have a higher glycolytic rate and reduced OXPHOS activity^[4]^. However, TRAP1 deletion did not impair other CSC-associated features, such as cell proliferation and chemoresistance to drugs affecting DNA polymerization. These included Gemcitabine, which inhibits cancer cell growth by inhibiting DNA synthesis and inducing apoptosis^[20]^, Oxaliplatin, which forms platinum complexes that bind to DNA^[21]^, Irinotecan, which inhibits topoisomerase I leading to double-strand DNA breaks^[22]^, and 5-fluorouracil, which disrupts DNA and RNA synthesis^[23]^. Therefore, the comparable effect of these compounds on wild-type and TRAP1-KO CSCs is likely attributable to the similarly low proliferative capacity of these two cell populations. Altogether, these data suggest that while TRAP1 is not essential for establishing the basic CSC phenotype, it contributes to specific aspects of their organization and function. Specifically, three crucial features are altered in TRAP1-KO CSCs: (i) the ability to form spherical aggregates; (ii) mitochondrial and cristae morphology; and (iii) ATP production and OCRs.

Regarding spheroid formation, we observed that TRAP1-KO CSCs were unable to form well-organized spheroids. Unlike control CSCs, which generated compact aggregates, TRAP1-deficient CSCs produced smaller and disordered structures even after prolonged culture. This result may be associated with the increased *CDH1* levels in TRAP1-knockout P cells, suggesting that the acquisition of a suspension phenotype^[24]^ may be slowed by the high basal levels of E-cadherin. Furthermore, we demonstrated the functional link between TRAP1 and CDH1 by supplementing the cell culture medium with succinate, thereby mimicking the effect of succinate dehydrogenase inhibition by TRAP1.

Succinate acts as an oncometabolite by inhibiting alpha-ketoglutarate-dependent dioxygenases, including histone demethylases that regulate the transcription of *CDH1*^[25]^. When we treated Panc1 P cells and TRAP1-KO P cells with succinate, *CDH1* transcription was selectively increased in TRAP1-KO cells. In contrast, it remained unchanged in TRAP1-expressing cells, where high succinate levels maintained via SDH inhibition are likely sufficient to enhance histone demethylation and the consequent *CDH1* transcription. As histone (de)methylation reactions are deregulated in most cancer types, where they tune the expression of a variety of important genes, future studies should investigate how TRAP1 regulates the broader transcriptional landscape in our pancreatic CSC model. Altogether, although direct metabolomic quantification of succinate levels was not performed, the consistent dose-dependent modulation of CDH1 mRNA levels upon succinate supplementation strongly supports the involvement of this metabolite in the TRAP1-mediated pathway.

TEM analysis revealed a profound mitochondrial remodeling in the absence of TRAP1. TRAP1-KO parental cells displayed elongated mitochondria with increased area and enlarged cristae. Upon de-differentiation, these mitochondria did not revert to the typical CSC phenotype; instead, they adopted an alternative organization characterized by increased fragmentation and thinner, more extended cristae, and tighter junctions. At the molecular level, neither TRAP1-knockout cell type showed changes in DRP1, OPA1, or MFN2 expression, whereas MFF and MFN1 levels were lower in TRAP1-KO P cells than in CSCs 2w TRAP1-KO. Despite the reduction in MFN1 levels, which typically impairs mitochondrial fusion, the loss of MFF exerts a dominant effect in TRAP1-KO P cells, shifting the mitochondrial dynamic equilibrium predominantly toward fusion. Conversely, CSCs 2w TRAP1-KO showed significantly increased MFF levels and similar levels of MFN1 compared to P cells, correlating with the lower mitochondrial aspect ratio detected via TEM.

These structural changes in mitochondria cristae likely underpin the metabolic profiles observed in our bioenergetic experiments. Consistently with TRAP1’s inhibitory effect on respiratory chain complexes, its deletion produced a marked shift toward oxidative metabolism. Specifically, TRAP1-KO cells, and more particularly TRAP1-KO CSCs, displayed a similar total ATP production rate but a significant shift towards OXPHOS. This was accompanied by enhanced maximal respiration, increased coupling efficiency, and reduced proton leak.

Taken together, these data suggest that the higher maximal respiration of TRAP1-KO CSCs is associated with the tightened cristae junctions observed by TEM. These findings indicate that TRAP1 may act both as a metabolic brake on mitochondrial respiration and as a regulator of mitochondrial structure. Indeed, cristae shape determines the assembly of respiratory chain supercomplexes, respiratory efficiency, and cellular growth^[18]^. Our results support the hypothesis of cooperation between TRAP1 and MICOS, a key complex in the inner mitochondrial membrane responsible for protein import and architectural modeling^[26,27]^.

Additionally, future experiments should analyze TRAP1 activity and its effects on client proteins, which may vary based on post-translational modifications^[28,29]^ or point mutations linked to particular pathological conditions^[30]^.

Despite these novel insights, some limitations must be acknowledged. First, the effects of TRAP1-KO in CSCs were based on the Panc1 cell line. While Panc1 is a well-established model, it may not fully recapitulate the heterogeneity of PDAC. Second, further validation using *in vivo* models, e.g., murine models, is warranted to determine whether TRAP1 ablation effectively reduces tumor-initiating capacity, metastatic potential, and resistance to chemotherapy agents. Third, while the *in vitro* spheroid model reproduces several features of the CSC niche, it does not fully capture the complexity of the tumor microenvironment, particularly interactions with stromal and immune components.

In conclusion, since metabolic plasticity is a hallmark of PDAC CSCs, targeting TRAP1 may destabilize CSC homeostasis, making these cells more vulnerable to metabolic inhibitors or combination therapies. However, as TRAP1 inhibition alone does not eliminate stemness traits or chemoresistance, it may need to be combined with agents that exploit the increased oxidative dependency of TRAP1-deficient CSCs or that target adhesion-related vulnerabilities. This work identifies TRAP1 as a key regulator of mitochondrial architecture, metabolic routing, and CDH1-dependent adhesion in PDAC CSCs. By linking TRAP1 to succinate-mediated transcriptional control and to cristae remodeling, our findings highlight new metabolic vulnerabilities that could be exploited therapeutically to overcome CSC-driven resistance and tumor recurrence in pancreatic cancer.

## DECLARATIONS

### Availability of data and materials

The datasets used and analyzed during the current study are available from the corresponding author on request.

### Copyright

© The Author(s) 2026.
