# Supplementary material for: TRAP1 ablation improves mitochondrial cristae and oxidative phosphorylation in pancreatic cancer stem cells
Source: Cancer Drug Resist. 2026 May 15;9:17. doi: 10.20517/cdr.2025.229 (PMC13244268; doi:10.20517/cdr.2025.229)
Supplement: Supplementary file 1 [file cdr-9-17-SupplementaryMaterials.pdf]

Supplementary Materials

TRAP1 ablation improves mitochondrial cristae and oxidative phosphorylation in pancreatic cancer stem cells

Giulia Ambrosini[1], Elisa Dalla Pozza[1], Ilaria Cristanini[1], Sara Vinco[1], Enrica Cappellozza[2], Barbara Cisterna[1], Claudio Laquatra[3], Andrea Rasola[3], Emanuela Bottani[2], Ilaria Dando[1]

[1]Department of Neurosciences, Biomedicine and Movement Sciences, University of Verona, Verona 37134, Italy.

[2]Department of Diagnostics and Public Health, University of Verona, Verona 37134, Italy.

[3]Department of Biomedical Sciences, University of Padova, Padova 35131, Italy.

Correspondence to: Prof. Ilaria Dando, Department of Neurosciences, Biomedicine and Movement Sciences, University of Verona, Verona 37134, Italy. E-mail: ilaria.dando@univr.it

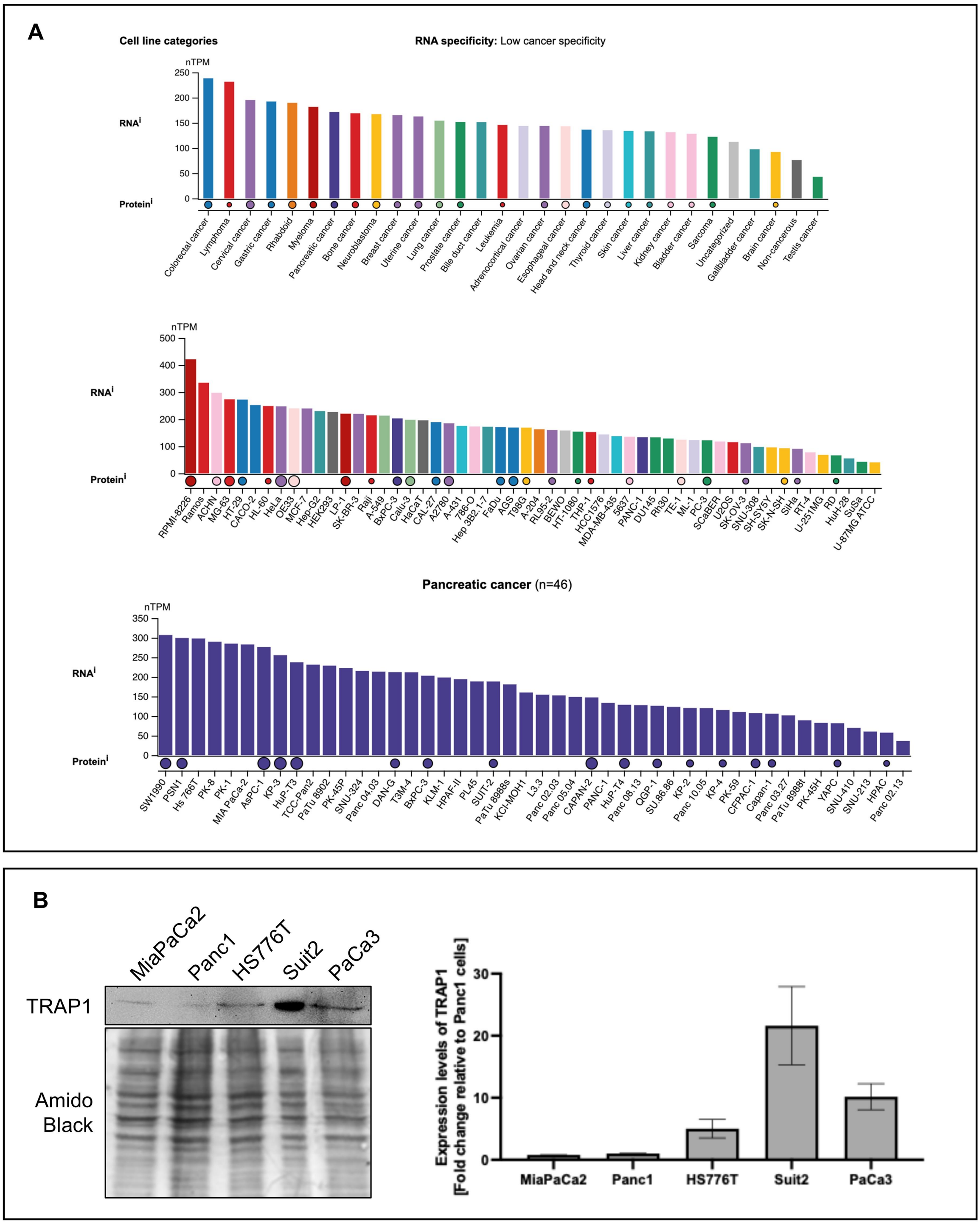

Supplementary Figure 1. (A) In the first graph, bioinformatic analysis of TRAP1 RNA expression levels across different cell categories, including both tumor and non-tumor cells; among these, pancreatic cancer ranks seventh in terms of TRAP1 expression. The subsequent panel shows TRAP1 RNA expression levels in individual cell lines, allowing identification of the pancreatic cancer cell line Panc1. The third panel, focused specifically on pancreatic cancer, highlights differences in TRAP1 expression levels among pancreas-specific cell lines. Data are obtained through statistical analysis and machine learning and are available in the Human Protein Atlas (www.proteinatlas.org). (B) Representative Western blot analysis of TRAP1 expression in five different PDAC cell lines, together with the densitometric analysis, on which the histograms represent the quantification of band intensity reported as fold change relative to Panc1 cells. Amido black is shown as the loading control.

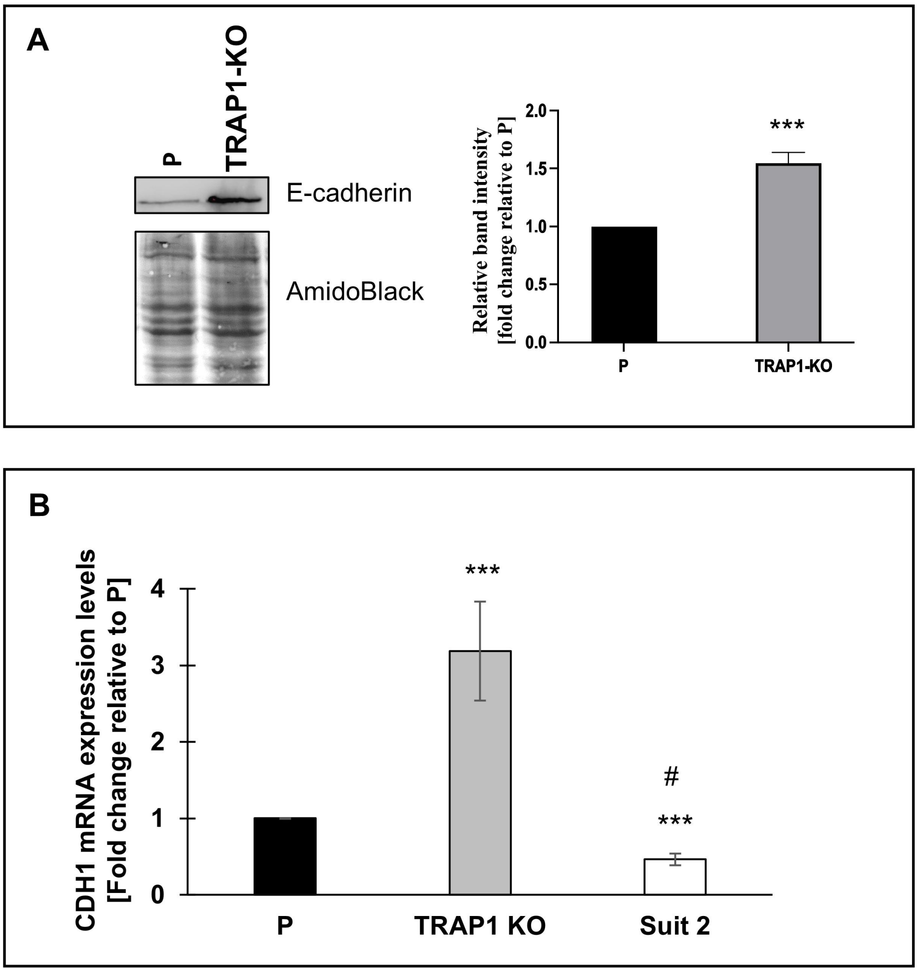

Supplementary Figure 2. (A) Representative Western blot analysis of E-cadherin expression in P and TRAP1-KO cells; the histogram represents the quantification of band intensity reported as fold change relative to P cells. Amido black is shown as the loading control. (B) qPCR analysis of *CDH1*in P, TRAP1-KO and Suit2 cell line. The values are reported as fold change relative to P cells. All values are the means (± SE) of at least three independent biological replicates. Statistical legend: $p < 0.05$ (*) indicated condition versus P cells, (#) indicated condition versus TRAP1-KO.

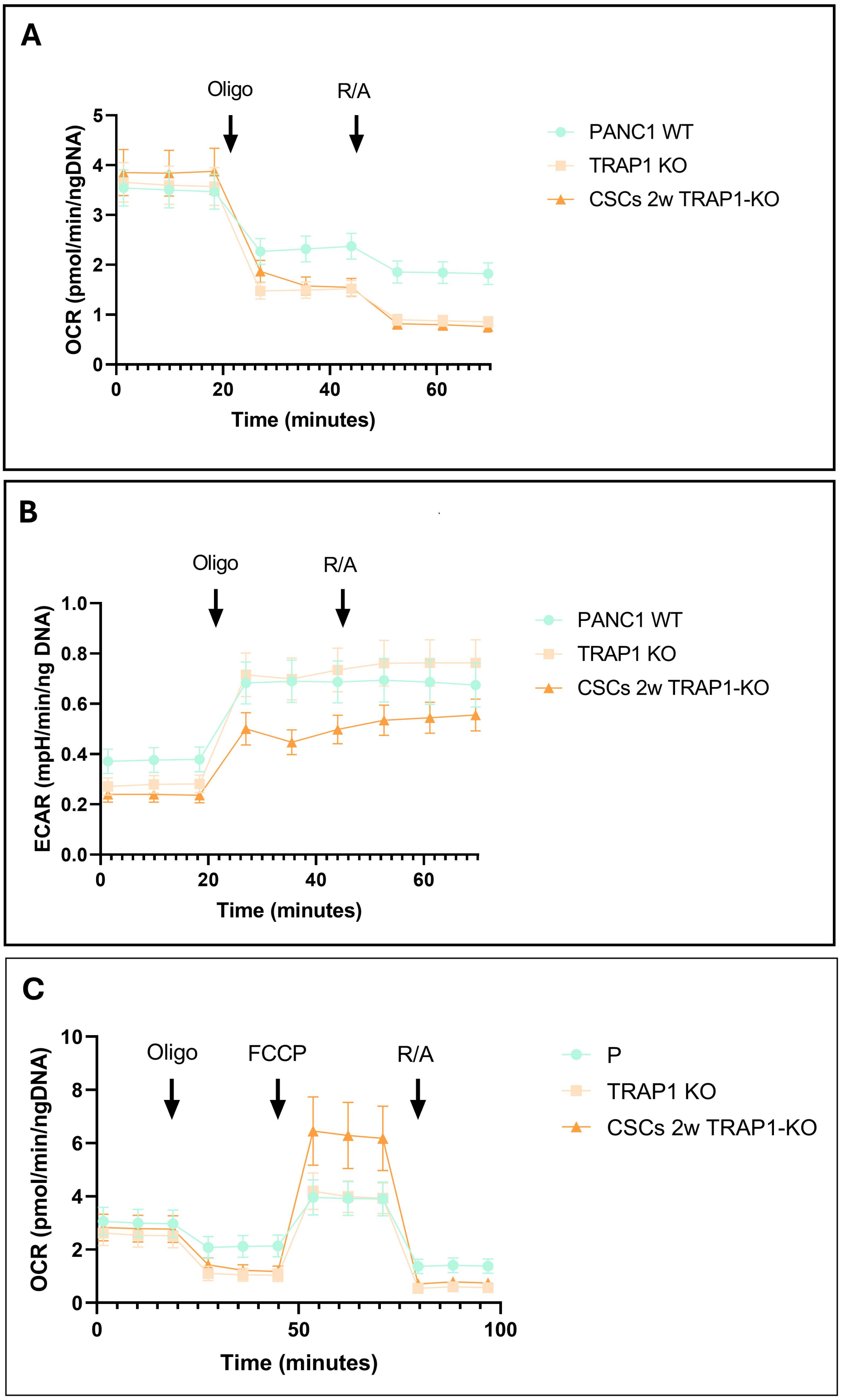

Supplementary Figure 3. Raw traces of (A) oxygen consumption rate (OCR) and (B) extracellular acidification rate (ECAR) measured by Seahorse XF ATP Rate Assay in P, TRAP1-KO, and CSCs 2w TRAP1-KO cells. Oligomycin (Oligo) and Rotenone/Antimycin A (R/A) were sequentially injected at the indicated time points. (C) Raw OCR traces measured by Seahorse XF Mito Stress Test in the same cell lines, following sequential injection of Oligo, FCCP, and R/A. For all panels, OCR (pmol/min/ngDNA) and ECAR (mpH/min/ngDNA) values are shown as a function of time (minutes). Data represent the mean ± SEM of three independent experiments. Quantitative parameters derived from the ATP rate assay and Mito Stress Test are reported in Figure 5A and 5B, respectively.

Supplementary Table 1. Analysis of doubling time of cultured cells

	Doubling Time (days)
P	1.8 ± 0.03
TRAP1-KO	1.8 ± 0.05
CSCs 2wk	3.8 ± 0.07 * #
CSCs 2wk TRAP1-KO	4.0 ± 0.04 * #
CSCs 4wk	3.5 ± 0.02 * #
CSCs 4wk TRAP1-KO	3.3 ± 0.30 * #
CSCs 8wk	7.2 ± 0.25 * #
CSCs 8wk TRAP1-KO	7.0 ± 1.06 * #

Statistical legend: $p < 0.05$ (*) versus P cells and (#) versus TRAP1-KO cells.
